# Health-related quality of life among people who inject drugs in Australia

**DOI:** 10.1007/s11136-023-03465-3

**Published:** 2023-06-23

**Authors:** Qinglu Cheng, Sahar Bajis, Evan Cunningham, Sophy T. F. Shih, Marcel Schulz, Alison D. Marshall, Natasha K. Martin, Alec Miners, Behzad Hajarizadeh, Virginia Wiseman, Gregory J. Dore, Jason Grebely

**Affiliations:** 1 The Kirby Institute, UNSW Sydney, Wallace Wurth Building, High Street, Kensington, Sydney, NSW 2052 Australia; 2 St Vincent’s Clinical School, UNSW Medicine, Sydney, NSW Australia; 3 Department of Clinical Pharmacology and Toxicology, St Vincent’s Hospital, Sydney, NSW Australia; 4 Centre for Social Research in Health, UNSW Sydney, Sydney, NSW Australia; 5 Division of Infectious Diseases and Global Public Health, University of California San Diego, La Jolla, CA USA; 6 Department of Health Services Research and Policy, London School of Hygiene and Tropical Medicine, London, UK; 7 Department of Global Health and Development, London School of Hygiene and Tropical Medicine, London, UK

**Keywords:** Health-related quality of life, EQ-5D-3L, People who inject drugs, Hepatitis C, Liver fibrosis

## Abstract

**Purpose:**

There is limited research on health-related quality of life (HRQoL) among people who inject drugs (PWID). We aimed to evaluate factors associated with HRQoL among a cohort of PWID in Australia.

**Methods:**

Participants were enrolled in an observational cohort study (the LiveRLife Study) between 2014 and 2018 at 15 sites in Australia. They provided fingerstick whole-blood samples for point-of-care HCV RNA testing and underwent transient elastography to assess liver disease. Participants completed the EQ-5D-3L survey at enrolment. Regression models were used to assess the impact of clinical and socioeconomic characteristics on the EQ-5D-3L scores.

**Results:**

Among 751 participants (median age, 43 years; 67% male), 63% reported injection drug use in the past month, 43% had current HCV infection, and 68% had no/mild liver fibrosis (F0/F1). The mean EQ-5D-3L and EQ-VAS scores were 0.67 and 62, respectively, for the overall study population. There was no significant difference in the EQ-5D-3L scores among people with and without recent injecting drug use (mean: 0.66 vs. 0.68, median: 0.73 vs. 0.78, P = 0.405), and among people receiving and not receiving opioid agonist therapy (mean: 0.66 vs. 0.68, median: 0.73 vs. 0.76, P = 0.215). Participants who were employed were found to have the highest mean EQ-5D-3L (0.83) and EQ-VAS scores (77). The presence of current HCV infection, liver fibrosis stage, and high-risk alcohol consumption had little impact on HRQoL.

**Conclusions:**

The study findings provide important HRQoL data for economic evaluations, useful for guiding the allocation of resources for HCV elimination strategies and interventions among PWID.

**Supplementary Information:**

The online version contains supplementary material available at 10.1007/s11136-023-03465-3.

## Plain English summary

Health-related quality of life (HRQoL) refers to a person’s wellbeing in physical, mental, and social domains of health. Few studies have investigated the HRQoL among people who inject drugs. This study attempted to fill the literature gap by measuring HRQoL using a questionnaire called EQ-5D-3L and examine factors associated with lower HRQoL. This study did find a lower HRQoL score among the study population, which is consistent with previous findings. However, EQ-5D-3L could not discriminate between participants with different stages of liver disease, nor between participants with and without hepatitis C infection. Future research using other quality of life measures is warranted to better understand the HRQoL among people who inject drugs.

## Introduction

Health-related quality of life (HRQoL) refers to a person’s wellbeing in physical, mental, and social domains of health [1]. People who inject drugs (PWID) experience a lower HRQoL than the general population due to factors including increased psychological distress, unstable housing, unemployment, a history of drug overdose, and poor oral health [2, 3]. PWID are also at high risk of hepatitis C virus (HCV) infection [4]. Previous studies among PWID have demonstrated little impact of current HCV infection on HRQoL, while awareness of HCV status is associated with lower HRQoL [5, 6]. However, there is limited evaluation of the impact of various characteristics on HRQoL among PWID. Accurate information on HRQoL among PWID is critical to inform health economic evaluations of interventions to enhance HCV testing and treatment.

There is increasing demand for cost-utility analysis, a type of economic evaluation, to provide economic evidence as to whether a new health intervention or health technology is worth the investment. An essential component of cost-utility analysis is health utility, usually derived from preference-based HRQoL instruments. Health utilities are used for calculating quality-adjusted life years (QALYs), a commonly used measure of health outcomes for comparing health interventions or technologies. Cost-utility studies have assessed the cost-effectiveness of screening and treating HCV infection for PWID, but these studies are limited by the use of utility weights of people who do not inject drugs and often lack of liver disease staging data [7–10]. Although there are studies that have evaluated health utility among PWID [6, 11], these studies have been limited by small sample sizes and the absence of health utility information stratified by important sub-group analyses, including HCV infection status, injecting drug use (history and recent), current opioid agonist treatment (OAT), and liver disease staging. The availability of detailed health utility estimates in various sub-populations is critical to inform mathematical modelling parameters for cost-utility analyses.

To address this gap in the literature, we evaluated HRQoL among PWID recruited from drug use treatment and needle and syringe program sites in Australia. We also estimated health utility weights for different sub-populations (including by HCV infection status, former/recent injection drug use, current OAT, and by liver disease stage). Lastly, we evaluated factors associated with HRQoL in this population.

## Methods

### Study design

LiveRLife is an observational cohort study assessing a community-based model of care integrating a liver health promotion campaign, point-of-care HCV testing and non-invasive liver fibrosis assessment with linkage to care and HCV treatment among people with a history of injection drug use [12, 13].

### Study sample/population

Participants were enrolled between 14 July 2014 and 22 February 2018 at 15 sites in three jurisdictions in Australia. Study recruitment was conducted through a network of drug and alcohol clinics, needle and syringe programs, a medically supervised injecting center, a community health clinic, and an Aboriginal and Medical Service. All participants provided written informed consent and the study protocol and amendments were approved by the Human Research Ethics Committee at St. Vincent's Hospital, Sydney (HREC/12/SVH/34).

Eligible participants in the LiverRLife study were at least 18 years old and self-reported a history of injection drug use. Exclusion criteria between 2014 – 2016 included currently or previously received HCV treatment, having received transient elastography assessment and/or liver biopsy assessment in the previous two years, and current pregnancy (due to a contraindication for transient elastography). The protocol was revised to remove exclusion criteria (except for current pregnancy) to the study procedures for participants recruited after 14 January 2016.

### Study assessments

Enrolment assessments included fingerstick whole-blood sample collection for point-of-care HCV RNA testing using Xpert^®^ HCV Viral Load Fingerstick assay, dried blood spot collection, self-reported behavioural survey on tablet computer, liver disease assessment, and clinical nurse assessment. Liver disease was assessed using transient elastography by FibroScan^®^, which has a lower and upper detection limit of 2.5 and 75 kPa, respectively. Fibrosis stages were defined by scores 2.5–7.4 (F0/1—no/mild fibrosis), 7.5–9.4 (F2—moderate fibrosis), 9.5–12.4 (F3—severe fibrosis) and ≥ 12.5 kPa (F4, cirrhosis) [14]. A liver stiffness measurement score is considered valid if a minimum of 10 valid readings, with at least a 60% success rate and an interquartile range of ≤ 30% of the median value, is taken.

The self-administered questionnaire collected information on demographics (age, gender, Aboriginal and Torres Strait Islander identity, employment status, education level, housing status), drug use history, incarceration history, previous HCV testing and treatment, self-reported HCV status, self-reported OAT status and alcohol consumption. Participants were asked if they had injected drugs in the past six months, and if they had, they were asked if they injected drugs in the past month. Recent injecting was defined as injecting drugs in the previous month. Stable housing was defined as living in a rented or owned house or flat. Alcohol consumption was assessed using the Alcohol Use Disorders Identification Test (AUDIT-C), a 3-item alcohol screen that can help to detect persons who are high risk alcohol drinkers or who have active alcohol use disorders [15]. The AUDIT-C is scored on a scale of 0–12 with scores of ≥ 4 in men and ≥ 3 in women considered as high-risk drinking [16, 17].

In addition to the behavioral survey, participants also completed the EuroQoL five-dimension three-level (EQ-5D-3L) survey at the enrollment visit. The EQ-5D-3L instrument is made up of two components: 1) the EQ-5D-3L descriptive system; and 2) the EQ visual analogue scale (EQ-VAS). The EQ-5D descriptive system measures current self-perceived health comprised of five questions covering five domains (mobility, self-care, ability to do usual activities, pain or discomfort, and anxiety/depression), each with three response levels (no, some, or extreme problems). The EQ-VAS involved having patients rate how much they viewed their current health on a vertical scale from 0 to 100 (worst to best health imaginable).

### Outcome measures

The primary outcome of interest was health utility. Participants’ responses to the EQ-5D-3L survey were converted into health utility scores by applying the Australian value set and scoring algorithm [18]. The utility score could range from − 0.217 to 1, where a score lower than zero refers to a health state worse than death, 0 for death and 1 for perfect health.

### Statistical analysis

Participants’ baseline data, collected at enrollment were used for analyses. Descriptive statistics including means, frequencies and percentages were used to summarize the data. Mann–Whitney U tests and Kruskal–Wallis tests were used for comparing the HRQoL scores between subgroups. The response distribution for each domain of EQ-5D-3L was tabulated by fibrosis stage to understand how the severity of liver disease may impact on different aspects of health (both physical and mental). Demographic and behavioral factors hypothesized to be associated with lower EQ-5D-3L utility scores and EQ-VAS scores included older age, male sex, housing instability, history of incarceration, recent injecting drug use, not receiving OAT, high risk alcohol consumption, current smoking (currently daily smoking and current less than daily smoking), self-reported HCV infection, positive HCV RNA test results, and severe liver fibrosis stage. Participants with missing or unknown demographic and behavioral characteristics were grouped to ‘missing’ or ‘unknown’ subgroups and still included in the statistical analysis.

Regression models were used to assess the impact of clinical and sociodemographic characteristics associated with EQ-5D-3L. As EQ-5D-3L scores in the LiveRLife study were found to have a ceiling effect (about 20% participants reported perfect health), a two-part model was applied to address the skewness presented in the data. In the first part of the model, a logistic regression model was used to predict the likelihood that participants reported full health. In the second part of the model, a generalized linear model (GLM) with the log link and gamma distribution was fit to EQ-5D-3L scores smaller than one to assess which factors would influence the HRQoL among PWID. Marginal effects were then generated from the combined model. Negative marginal effect indicated poorer HRQoL, whereas positive marginal effect indicated better HRQoL. Association between clinical and sociodemographic factors and EQ-5D-3L utility scores were initially analyzed in unadjusted univariate analyses. Variables with a p < 0.05 at the unadjusted level or known clinical significance were considered for adjusted multivariate models. The two-part modelling was done using the Stata twopm command [19]. For all analyses, statistically significant differences were assessed at a 0.05 level; p-values were two-sided. All analyses were performed using Stata v15.0.

## Results

Overall, 751 individuals were enrolled into the LiveRLife study (Table 1). The median age of the cohort was 43 years (IQR: 36–51) and 67% were male. Nearly one-third of participants (29%) completed high school or higher education, 85% received government assistance as the main source of income, and 8% were employed. Most participants (72%) reported injection drug use in the past six months and 63% injected in the last month. Current HCV infection was detected in 43% of the study population and 68% of participants had no/mild liver disease. There were 44 participants who reported currently receiving HCV treatment. We did not exclude this group of participants from further analysis as we observed that current HCV treatment had no impact on the EQ-5D-3L scores (Appendix Tables A1 and A2).

**Table 1:** Baseline characteristics (n = 751)

Characteristics	n (%)
Age, median (IQR)	43 (36–51)
Age groups	
18–35	168 (22)
36–50	392 (52)
≥ 51	191 (25)
Sex	
Male	503 (67)
Female	242 (32)
Transgender	6 (< 1)
Aboriginal/Torres Strait Islander identity	
Yes	181 (24)
No	563 (75)
Unknown	7 (1)
Completed high school or higher education	
Yes	214 (29)
No	537 (71)
Main source of income	
No income	21 (3)
Full-time/part-time/casual employment	58 (8)
Government assistance	637 (85)
Other	35 (4)
Housing	
Stable	522 (70)
Unstable	229 (30)
Incarceration	
Never	355 (47)
Ever (not in past 12 months)	248 (20)
In past 12 months	148 (33)
Injected drugs in past 6 months	
Yes	541 (72)
No	210 (28)
Injected drugs in past month	
Yes	475 (63)
No	276 (37)
Hazardous alcohol consumption (AUDIT-C)^a^	
Never drinks	331 (44)
Low risk male/female	160 (21)
High risk male/female	254 (34)
Smoking stats	
Never	35 (5)
Previous	74 (10)
Current	642 (85)
Opioid agonist therapy	
Current	517 (69)
Previous, not current	91 (12)
Never	143 (19)
Self-reported HCV infection	
Negative	222 (29)
Positive	396 (53)
Unknown	133 (18)
Previous/current HCV treatment^b^	81 (20)
HCV RNA test result	
Negative	372 (50)
Positive	323 (43)
Invalid/missing	56 (7)
FibroScan^®^ liver disease staging	
F0/F1—no/mild fibrosis	514 (68)
F2—moderate fibrosis	85 (11)
F3—severe fibrosis	44 (6)
F4—cirrhosis	63 (8)
Invalid	34 (5)
Missing	11 (1)

^a^Transgender excluded from AUDIT-C (n = 6)

^b^Among participants reporting having HCV infection (n = 396)

All the 751 participants completed the EQ-5D-3L questionnaire. The EQ-5D-3L and EQ-VAS scores by baseline characteristics are summarized in Table 2. The mean EQ-5D-3L score and EQ-VAS score for the overall population were 0.67 and 62, respectively. The mean EQ-5D-3L scores were the highest among participants who were employed (0.83) compared to those receiving government assistance (0.73). There were no significant differences among people with and without recent injecting drug use (mean: 0.66 vs. 0.68, median: 0.73 vs. 0.78, P = 0.405), people receiving and not receiving OAT (mean: 0.66 vs. 0.68, median: 0.73 vs. 0.76, P = 0.215), people self-reporting HCV infection and no HCV infection (mean: 0.67 vs. 0.66, median: 0.73 vs. 0.73, P = 0.716) and people with and without confirmed current HCV infection (mean: 0.67 vs. 0.67, median: 0.73 vs. 0.73, P = 0.964). For EQ-VAS scores, there was no significant difference among people with and without recent injecting drug use (mean: 61 vs. 66, median: 70 vs. 70, P = 0.246), people receiving and not receiving OAT (mean: 61 vs. 62, median: 68 vs. 70, P = 0.470), and people with and without current HCV infection (mean: 61 vs. 63, median: 70 vs. 70, P = 0.444). Again, participants who were employed had the highest EQ-VAS scores compared to those receiving government assistance (mean: 77 vs. 61, median: 80 vs. 65, P < 0.001). There was also a significant difference in the EQ-VAS scores among people with and without stable housing (mean: 64 vs. 57, median: 70 vs. 60, P = 0.006), people who were never and current smokers (mean: 70 vs. 61, median: 75 vs. 65, P = 0.007) and people self-reporting HCV infection and no HCV infection (mean: 59 vs 65, median: 65 vs. 70, P = 0.002). For people who tested positive for HCV, those who were currently receiving treatment reported a higher mean EQ-VAS score than those not receiving treatment (Appendix Table A1, 73 vs. 62).

**Table 2:** Health-related quality of life by baseline characteristics

	n	Mean EQ-5D (SD)	Median EQ-5D (IQR)	p value*	Mean VAS (SD)	Median VAS (IQR)	p value*
Overall	751	0.67 (0.27)	0.73 (0.50–0.83)		62 (26)	70 (50–80)	
Age groups				0.688			0.643
18–35	168	0.67 (0.28)	0.74 (0.50–0.83)		62 (26)	63 (50–80)	
36–50	392	0.67 (0.27)	0.73 (0.50–0.80)		61 (26)	68 (50–80)	
≥ 51	191	0.65 (0.27)	0.73 (0.45–0.83)		64 (24)	70 (50–80)	
Sex^a^				0.767			0.261
Male	509	0.67 (0.27)	0.73 (0.50–0.83)		62 (26)	70 (50–80)	
Female	242	0.67 (0.27)	0.73 (0.50–0.80)		60 (25)	60 (50–80)	
Aboriginal/Torres Strait Islander identity^b^				0.510			0.908
Yes	181	0.66 (0.28)	0.73 (0.50–0.83)		62 (25)	60 (50–80)	
No	563	0.66 (0.27)	0.73 (0.50–0.83)		61 (28)	70 (50–80)	
Completed high school or higher education				0.126			0.068
Yes	214	0.69 (0.25)	0.75 (0.53–0.83)		65 (24)	70 (50–80)	
No	537	0.66 (0.25)	0.73 (0.50–0.80)		61 (26)	65 (50–80)	
Main source of income				< 0.001			< 0.001
No income	21	0.75 (0.23)	0.80 (0.71–1.00)		64 (22)	60 (50–90)	
Full-time/part-time/casual employment	58	0.82 (0.22)	0.83 (0.75–1.00)		77 (14)	80 (70–90)	
Government assistance	637	0.66 (0.27)	0.73 (0.50–0.80)		61 (26)	65 (50–80)	
Other	35	0.57 (0.29)	0.66 (0.24–0.80)		57 (26)	60 (40–75)	
Housing				0.067			0.006
Stable	522	0.68 (0.27)	0.73 (0.50–0.83)		64 (25)	70 (50–80)	
Unstable	229	0.64 (0.27)	0.73 (0.50–0.80)		57 (27)	60 (40–80)	
Incarceration				0.563			0.525
Never	248	0.67 (0.27)	0.74 (0.50–0.81)		63 (26)	70 (50–80)	
Ever (not in past 12 months)	355	0.66 (0.26)	0.73 (0.50–0.80)		61 (25)	66 (50–80)	
In past 12 months	148	0.67 (0.29)	0.80 (0.50–0.83)		61 (27)	68 (49–80)	
Recency of drug injecting				0.405			0.246
Ever, but not in past 6 months	210	0.68 (0.28)	0.75 (0.52–0.83)		62 (27)	70 (49–85)	
None in the past month	66	0.68 (0.28)	0.78 (0.50–0.83)		66 (21)	70 (50–80)	
Injected in past month	475	0.66 (0.26)	0.73 (0.50–0.80)		61 (26)	70 (50–80)	
Hazardous alcohol consumption (AUDIT-C)^c^				0.529			0.085
High risk male/female	254	0.66 (0.28)	0.72 (0.50–0.80)		59 (28)	60 (40–80)	
Low risk male/female	160	0.69 (0.24)	0.73 (0.59–0.80)		64 (23)	70 (50–80)	
Never drinks	331	0.67 (0.28)	0.73 (0.50–0.83)		63 (26)	70 (50–80)	
Smoking status				0.050			0.007
Never	35	0.75 (0.23)	0.80 (0.68–0.84)		70 (23)	75 (51–85)	
Previous	74	0.69 (0.29)	0.73 (0.45–1.00)		68 (25)	75 (50–85)	
Current	642	0.66 (0.27)	0.73 (0.50–0.80)		61 (26)	65 (50–80)	
Current opioid agonist therapy				0.215			0.470
Yes	517	0.66 (0.26)	0.73 (0.50–0.80)		61 (26)	68 (50–80)	
No	234	0.68 (0.28)	0.76 (0.50–0.83)		62 (27)	70 (50–80)	
Self-reported HCV infection				0.716			0.002
Yes	396	0.67 (0.27)	0.73 (0.50–0.83)		59 (27)	65 (40–80)	
No	222	0.66 (0.27)	0.73 (0.45–0.83)		65 (23)	70 (50–80)	
HCV RNA test result				0.964			0.444
Negative	372	0.67 (0.28)	0.73 (0.50–0.83)		63 (26)	70 (50–80)	
Positive	323	0.67 (0.26)	0.73 (0.50–0.80)		61 (26)	70 (50–80)	
Missing	56	0.65 (0.30)	0.73 (0.46–0.80)		59 (27)	60 (40–80)	
FibroScan^®^ liver disease staging				0.046			0.075
F0/F1—no/mild fibrosis	514	0.68 (0.27)	0.75 (0.50–0.83)		63 (26)	70 (50–80)	
F2/3—moderate/severe	129	0.63 (0.25)	0.68 (0.45–0.80)		59 (25)	60 (49–80)	
F4—cirrhosis	63	0.67 (0.28)	0.73 (0.45–1.00)		65 (22)	70 (50–80)	
Invalid score/missing	45	0.61 (0.32)	0.66 (0.38–0.80)		55 (28)	50 (40–80)	

*IQR* interquartile range

^a^Trangender combined with male (n = 6)

^b^Participants responding as unknown are excluded (n = 7

^c^n = 6 excluded

*Mann–Whitney U tests and Kruskal–Wallis tests were used to compare EQ-5D-3L and EQ-VAS scores between subgroups

**Fig. 1:**
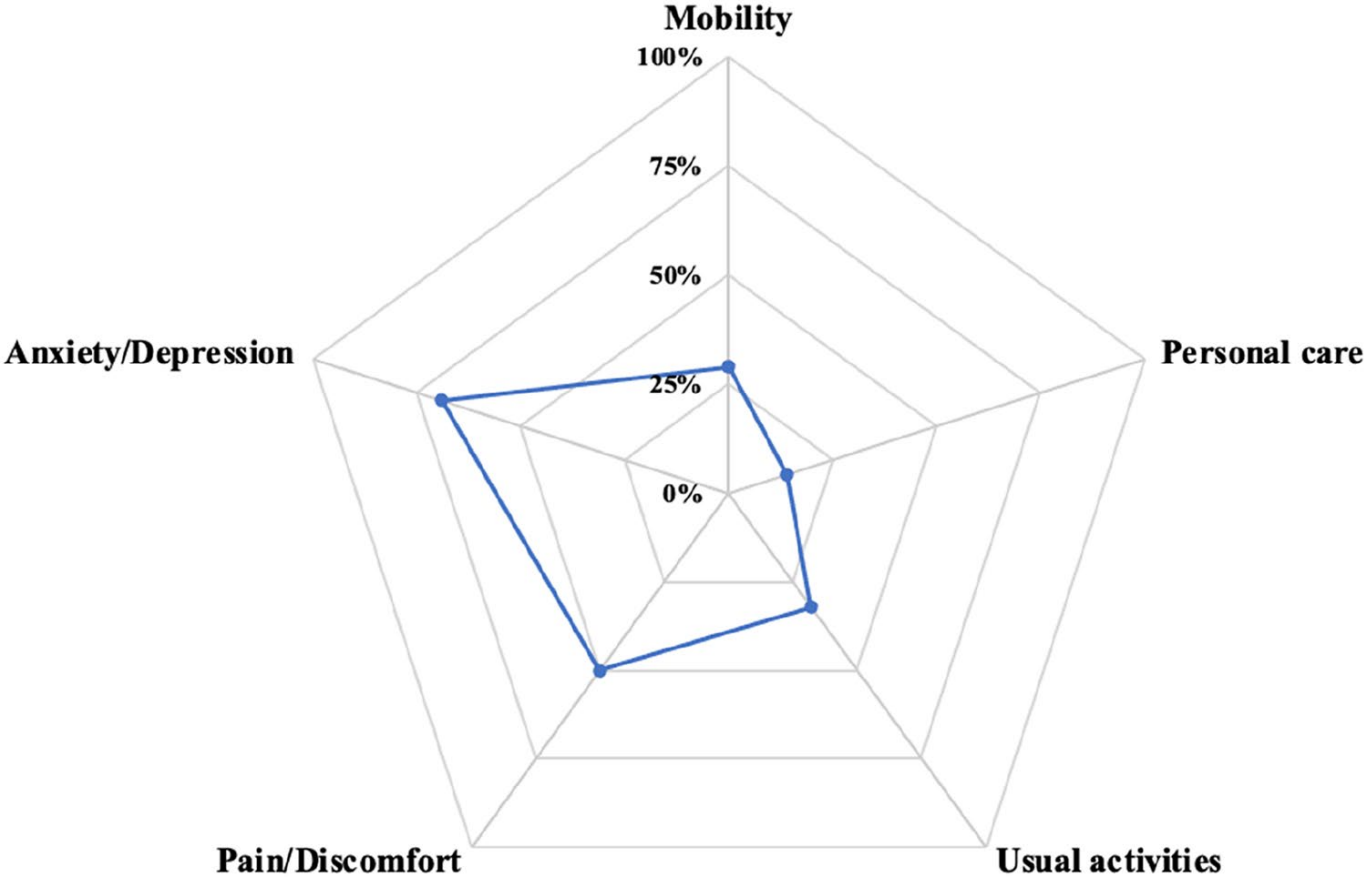
Proportion of responses indicating “some problems” or “severe problems” in each EQ-5D-3L domain in the overall study population

**Fig. 2:**
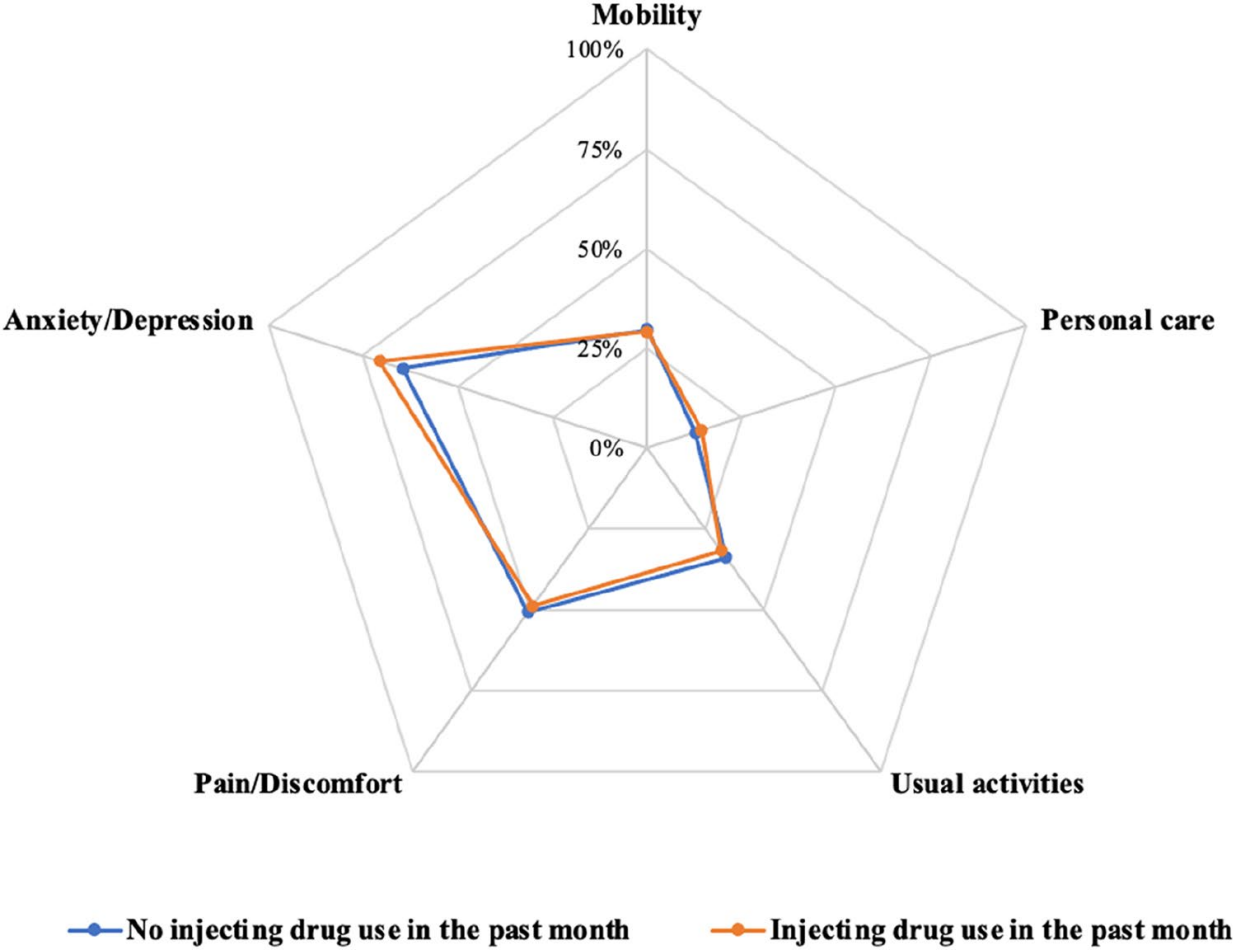
Proportion of responses indicating “some problems” or “severe problems” in each EQ-5D-3L domain by recent drug injection status

Overall, most people had no problems with mobility (71%), personal care (86%) and usual activities (68%) (Table 3). Nearly half (49%) of the study population were living with moderate or severe pain/discomfort (Fig. 1). Sixty-nine percent of participants reported they were extremely or moderately anxious or depressed. For self-care, usual activities and pain/discomfort domains, there was a statistically significant difference in the distribution of EQ-5D-3L responses by liver disease stage. Among people with significant liver fibrosis (≥ F2), there was an increased proportion of participants reporting issues with mobility and usual activities. There was little impact of liver disease stage on mobility and anxiety/depression domains. The proportions of responses indicating “some problems” or “severe problems” in each EQ-5D-3L domain were similar among people with and without recent injecting drug use (Fig. 2).

**Table 3:** EQ-5D-3L and EQ VAS health status classifications by FibroScan liver disease stage

EQ-5D-3L	Total population n = 751	FibroScan liver disease staging			
		F0/1	F2-F3	F4	p value
Mobility					0.185
I have no problems in walking around	530 (71%)	380 (74%)	85 (66%)	41 (65%)	
I have some problems in walking around	211 (28%)	127 (25%)	42 (33%)	22 (35%)	
I am confined to bed	10 (1%)	7 (1%)	2 (2%)	0 (0%)	
Personal care					0.029
I have no problems with personal care	648 (86%)	447 (87%)	107 (83%)	59 (94%)	
I have some problems washing or dressing myself	94 (13%)	64 (12%)	19 (15%)	2 (3%)	
I am unable to wash or dress myself	9 (1%)	3 (1%)	3 (2%)	2 (3%)	
Usual activities					0.018
I have no problems with performing my usual activities	507 (68%)	362 (70%)	75 (58%)	41 (65%)	
I have some problems with performing my usual activities	226 (30%)	140 (27%)	53 (41%)	19 (30%)	
I am unable to perform my usual activities	18 (2%)	12 (2%)	1 (1%)	3 (5%)	
Pain/discomfort					0.042
I have no pain or discomfort	379 (50%)	278 (54%)	54 (42%)	28 (44%)	
I have moderate pain or discomfort	282 (38%)	179 (35%)	61 (47%)	24 (38%)	
I have extreme pain or discomfort	90 (12%)	57 (11%)	14 (11%)	11 (17%)	
Anxiety/depression					0.292
I am not anxious or depressed	238 (32%)	168 (33%)	32 (25%)	25 (40%)	
I am moderately anxious or depressed	366 (49%)	248 (48%)	70 (54%)	28 (44%)	
I am extremely anxious or depressed	147 (20%)	98 (19%)	27 (21%)	10 (16%)	
EQ VAS, median (IQR)	70 (50, 80)	70 (50, 80)	60 (49, 80)	70 (40, 80)	0.157

p values chi-square test or Fisher’s exact test depending on expected cell frequencies; p value for EQ-VAS by Kruskal–Wallis test

Table 4 presents the relationship between baseline characteristics and EQ-5D-3L scores using the unadjusted two-part model. Compared to those with “no income”, earning income from government assistance (marginal effect = - 0.099, P = 0.049) and earning income from other sources (marginal effect = - 0.183, P = 0.012) were associated with significantly lower EQ-5D-3L scores. Compared to no smokers, a current smoker reported significantly lower EQ-5D-3L scores (marginal effect = -0.089, P = 0.017). In the adjusted analysis (Table 5), the same factors were associated with significantly lower EQ-5D-3L scores: earning income from government assistance (marginal effect = -0.098, P = 0.049), earning income from other sources (marginal effect = -0.192, P = 0.009) and being a current smoker (marginal effect = -0.084, P = 0.019). Results of the two-part model by recent drug injection status are presented in Appendix Tables A3, A4 and A5. For participants who injected drugs in the past month, factors associated with significantly lower EQ-5D-3L scores included earning income from other sources (marginal effect = -0.188, P = 0.030) and being a current smoker (marginal effect = -0.094, P = 0.043). Among participants who did not inject drugs in the past month, baseline characteristics had little impact on the EQ-5D-3L scores.

**Table 4:** Unadjusted univariate analysis of factors associated with EQ-5D-3L scores (using two-part model)

	Marginal effect	95% CI	p value*
Age groups			0.717
18–35	–		
36–50	− 0.004	− 0.053, 0.046	0.888
≥ 51	− 0.021	− 0.077, 0.035	0.460
Sex			0.807
Male	–		
Female	− 0.003	− 0.044, 0.038	0.889
Aboriginal/Torres Strait Islander ethnicity			0.242
No	–		
Yes	− 0.010	− 0.057, 0.037	0.674
Completed high school or higher education			0.123
No	–		
Yes	0.033	− 0.008, 0.074	0.113
Main source of income			0.000
No income	–		
Full-time/part-time/casual employment	0.070	− 0.041, 0.181	0.215
Government assistance	− 0.099	− 0.198, 0.000	0.049
Other	− 0.183	− 0.325, − 0.040	0.012
Housing			0.259
Stable	–		
Unstable	− 0.035	− 0.077, 0.008	0.110
Incarceration			0.927
Ever (not in past 12 months)	–		
In past 12 months	0.008	− 0.044, 0.061	0.759
Never	0.005	− 0.038, 0.049	0.810
Recency of drug injecting			0.914
Ever, but not in past 6 months	–		
None in the past month	− 0.015	− 0.058, 0.029	0.515
Injected in past month	0.000	− 0.073, 0.073	0.997
Hazardous alcohol consumption (AUDIT-C)			0.223
Never drinks	–		
Low risk male/female	0.027	− 0.021, 0.074	0.271
High risk male/female	− 0.010	− 0.055, 0.035	0.658
Smoking stats			0.024
Never			
Previous	− 0.063	− 0.160, 0.034	0.203
Current	− 0.089	− 0.163, 0.016	0.017
Current opioid agonist therapy			0.097
No	–		
Yes	− 0.018	− 0.060, 0.024	0.409
HCV RNA test result			0.823
Negative	–		
Positive	0.004	− 0.036, 0.044	0.835
Missing	− 0.022	− 0.100, 0.056	0.575
Self-reported HCV status			0.591
No	–		
Yes	0.008	− 0.037, 0.053	0.718
Unknown	− 0.007	− 0.063, 0.050	0.812
FibroScan^®^ liver disease staging			0.107
F0/F1—no/mild fibrosis	–		
F2/3	− 0.048	− 0.099, 0.003	0.064
F4—cirrhosis	− 0.012	− 0.087, 0.064	0.761
Invalid score/missing	− 0.071	− 0.166, 0.024	0.143

*The overall p-values for variables with multiple categories were derived from Wald tests, using Stata function *testparm*

**Table 5:** Adjusted multivariate analysis of factors associated with EQ-5D-3L scores (using two-part model)

	Marginal effect	95% CI	p value*
Main source of income			0.000
No income	–		
Full-time/part-time/casual employment	0.065	− 0.045, 0.174	0.246
Government assistance	− 0.098	− 0.195, 0.000	0.049
Other	− 0.192	− 0.336, − 0.049	0.009
Smoking stats			0.031
Never			
Previous	− 0.071	− 0.165, 0.022	0.136
Current	− 0.084	− 0.154, 0.014	0.019
HCV RNA test result			0.735
Negative	–		
Positive	0.012	− 0.028, 0.052	0.560
Missing	− 0.018	− 0.094, 0.059	0.654
FibroScan^®^ liver disease staging			0.057
F0/F1—no/mild fibrosis	–		
F2/3	− 0.045	− 0.096, 0.006	0.082
F4—cirrhosis	− 0.024	− 0.103, 0.056	0.562
Invalid score/missing	− 0.079	− 0.174, 0.016	0.103

The overall p-values for variables with multiple categories were derived from Wald tests, using Stata function *testparm*

## Discussion

This study evaluated HRQoL and associated factors among a cohort of PWID in Australia. People in this study had little problems with mobility, self-care, and usual activities, but a large proportion experienced pain/discomfort and anxiety/depression. Unemployment, unstable housing, and current smoking were associated with lower HRQoL. Current HCV infection, liver fibrosis disease stage, recent injecting drug use, current OAT, and high-risk alcohol consumption had little impact on HRQoL. These data provide key information on HRQoL among PWID to guide future economic evaluation studies as well as clinical practice and policy.

The mean EQ-5D-3L (0.67) and EQ-VAS scores (62) were much lower in this sample than those elicited from the general population in Australia (0.91 and 79, respectively) [20]. This supports the existing evidence that PWID usually have impaired HRQoL compared to the general population [2, 3, 5, 21]. The mean and median EQ-5D-3L scores reported in this study are consistent with two previous studies that directly elicited health utility values from PWID using EQ-5D-3L [6, 11]. But it should be noted that participants in the Gormley et al. study, had received HCV treatment and achieved cure [11], while our study population included PWID with and without current HCV infection and the majority did not have a history of treatment. For people who inject drugs with current HCV infection (HCV RNA detectable), our study produced a similar median EQ-5D-3L score (0.73) to a study of people who inject drugs in Scotland (0.69) [6]. In the study by McDonald et al. [6], people testing positive for HCV who believed that they were negative reported higher EQ-5D-3L scores than those aware of their positive HCV status (0.74 vs. 0.66). Similarly, in a study by Dalgard et al. [5], among people with chronic HCV infection and who inject drugs, those who were aware of their infection had lower HRQoL scores across several SF-36 dimensions (general health, physical functioning, physical role, and vitality) than those unaware of their infection. Conversely, HCV RNA negative participants, who believed they were infected, scored lower in general health compared to those who did not believe they were infected [5]. In our study, participants who self-reported HCV infection also reported significantly lower EQ-VAS scores than those who believed they were not infected. Therefore, awareness of HCV is likely to be an important determinant of self-reported health. Further counselling and educational efforts are needed to alleviate fears and concerns about the impacts of current HCV infection in the context of the availability of highly effective and tolerable direct-acting antiviral treatments. However, the awareness of HCV had no significant impact on the EQ-5D-3L utility score, indicating that the awareness of HCV might influence aspects of health other than the five domains in the EQ-5D-3L.

We hypothesised that participants with current HCV and significant liver fibrosis (≥ F2) would experience poorer HRQoL than those with milder fibrosis (F0/F1). However, we observed that EQ-5D-3L and EQ-VAS could not discriminate between participants with different fibrosis stages. Participants with cirrhosis (F4) reported higher health utility scores than those with moderate fibrosis (F2/3), which is in contrast with findings from other studies where cirrhosis is associated with lower health utility scores compared to those with more mild liver disease among people with chronic HCV infection [22, 23]. It is possible that the small sample size of participants with advanced liver disease may have precluded the ability to discriminate differences in health utility by liver disease stage. Further, it is plausible that people with advanced liver diseases felt more supported through engagement with healthcare, which had a positive impact on their HRQoL. It is also possible that the EQ-5D-3L scale might not be sensitive enough to capture quality of life changes related to advanced liver disease progression among PWID. As EQ-5D-3L is a generic HRQoL instrument, it may fail to include some features specific to PWID, such as housing instability [24].

As consistent with previous studies [21, 25, 26], gender was not found statistically significantly associated with HRQoL in our study population. In this analysis, participants who were employed and those who had stable housing reported higher mean and median HRQoL scores. However, in the two-part model, these two factors were no longer statistically significant. This is different to findings by Scott et al. [2], which demonstrated being employed was significantly correlated with an increase in personal wellbeing index (PWI) scores, while moving into unstable accommodation was associated with declines in PWI scores among PWID [2]. It is likely that factors other than those collected by our study may have played a role in determining the HRQoL among the study population. Evidence on the impact of OAT on HRQoL is also mixed. While improvements in HRQoL in the long term were observed for some populations [27, 28], other studies have reported immediate increase in HRQoL but then diminishing effects and even deterioration in HRQoL [29–31]. Our study did not find any significant impact of OAT on EQ-5D-3L scores, probably because the study population was already engaged with health services and effect of OAT on HRQoL might have diminished at the time of enrolment.

A major strength of our study is that we have addressed a major evidence gap by describing health utility information stratified by important sub-groups including HCV infection, recent injecting drug use and liver disease stages. This study estimated the heath utility scores among PWID who were HCV negative, and also demonstrated the marginal impact of HCV on HRQoL among those with relatively mild diseases. These data are important for future mathematical modelling and cost-effectiveness analyses to evaluate interventions to enhance HCV testing and treatment among PWID. The economic evidence generated by mathematical modelling and cost-effectiveness analyses will in turn inform practice and policies about what interventions should be integrated into service delivery and into national and jurisdictional strategies.

One limitation of this study is that the participants represent a population already engaged in health services tailored for PWID, and thus, our sample may not be representative of the broader population of PWID. As such, the health utility estimates may be an overestimate of the HRQoL experienced among people who have recently injected drugs but do not attend health services. However, it is encouraging that there was little difference in EQ-5D-3L and EQ-VAS scores among people with and without recent injecting drug use in this study. Another limitation is the use of EQ-5D-3L instrument to measure participants’ HRQoL. Although EQ-5D-3L has high validity for the general population and several diseases [32–36], it has not been validated among PWID. The EQ-5D-3L instrument was chosen for the LiveRLife study because it was the best available alternative at that time. But the domains featured by EQ-5D-3L may not be adequate to capture the changes in HRQoL observed by HCV-specific measures such as HCV-PRO [37, 38]. Recent evidence also showed that EQ-5D-3L was less suited to detect smaller changes in health in PWID, compared to SF-6D, independent of if current health state was good or bad [39]. Future research is needed to better validate health utility measures in this population. In addition, data on other non-HCV related co-morbidities/liver disease such as fatty liver disease were not collected. In our study, around 6% of participants had invalid liver disease staging scores. This is due to the lack of large-size probe to assess liver disease in people who are overweight/obese. Although those who are overweight are likely to have poorer HRQoL [40], given the small number of participants with invalid FibroScan^®^ scores, the impact on the overall findings should be minimal. Finally, this study analysed data collected between 2014 and 2018. Although it is not anticipated that there have been any changes to the clinical practice which would have led to a major improvement in HRQoL among PWID, further research using more recent data is warranted to investigate HRQoL among this population.

In conclusion, a lower HRQoL was observed among PWID compared with the general population. Employment and stable housing were associated with better HRQoL. However, EQ-5D-3L and EQ-VAS could not discriminate between participants with different stages of liver disease. Further research is needed to identify more suitable tools to measure and better understand HRQoL among PWID. This will inform future health economic analyses for identifying optimal interventions to facilitate HCV elimination globally.

### Supplementary Information

Below is the link to the electronic supplementary material.

Supplementary file1 (DOC 32 KB)

## Data Availability

This publication involved information collected from consenting individuals. Data used for this research cannot be deposited on servers other than those approved by Ethics Committees. This publication has used highly sensitive health information through the collection of survey data. All identifying information, including full name, has been anonymized under strict privacy regulations. Except in the form of conclusions drawn from the data, researchers do not have permission to disclose any data to any person other than those authorized for the research project.

## References

[1] Karimi M, Brazier J (2016). Health, health-related quality of life, and quality of life: What is the difference?. PharmacoEconomics.

[2] Scott N, Carrotte ER, Higgs P, Stoové MA, Aitken CK, Dietze PM (2017). Longitudinal changes in personal wellbeing in a cohort of people who inject drugs. PLoS ONE.

[3] Truong A, Higgs P, Cogger S, Jamieson L, Burns L, Dietze P (2015). Oral health-related quality of life among an Australian sample of people who inject drugs. Journal of Public Health Dentistry.

[4] Grebely J, Larney S, Peacock A, Colledge S, Leung J, Hickman M, Vickerman P, Blach S, Cunningham EB, Dumchev K, Lynskey M, Stone J, Trickey A, Razavi H, Mattick RP, Farrell M, Dore GJ, Degenhardt L (2019). Global, regional, and country-level estimates of hepatitis C infection among people who have recently injected drugs. Addiction.

[5] Dalgard O, Egeland A, Skaug K, Vilimas K, Steen T (2004). Health-related quality of life in active injecting drug users with and without chronic hepatitis C virus infection. Hepatology.

[6] McDonald SA, Hutchinson SJ, Palmateer NE, Allen E, Cameron SO, Goldberg DJ, Taylor A (2013). Decrease in health-related quality of life associated with awareness of hepatitis C virus infection among people who inject drugs in Scotland. Journal of Hepatology.

[7] van Santen DK, de Vos AS, Matser A, Willemse SB, Lindenburg K, Kretzschmar MEE, Prins M, de Wit GA (2016). Cost-**effectiveness of hep**atitis C treatment for people who inject drugs and the impact of the type of epidemic; Extrapolating from Amsterdam, the Netherlands. PLoS ONE.

[8] Visconti AJ, Doyle JS, Weir A, Shiell AM, Hellard ME (2013). Assessing the cost-effectiveness of treating chronic hepatitis C virus in people who inject drugs in Australia. Journal of Gastroenterology and Hepatology.

[9] Martin NK, Hickman M, Miners A, Hutchinson SJ, Taylor A, Vickerman P (2013). Cost-effectiveness of HCV case-finding for people who inject drugs via dried blood spot testing in specialist addiction services and prisons. British Medical Journal Open.

[10] Barbosa C, Fraser H, Hoerger TJ, Leib A, Havens JR, Young A, Kral A, Page K, Evans J, Zibbell J, Hariri S, Vellozzi C, Nerlander L, Ward JW, Vickerman P (2019). Cost-effectiveness of scaling-up HCV prevention and treatment in the United States for people who inject drugs. Addiction.

[11] Gormley MA, Akiyama MJ, Rennert L, Howard KA, Norton BL, Pericot-Valverde I, Muench S, Heo M, Litwin AH (2022). Changes in health-related quality of life for hepatitis C virus-infected people who inject drugs while on opioid agonist treatment following sustained virologic response. Clinical Infectious Diseases.

[12] Bajis S, Grebely J, Cooper L, Smith J, Owen G, Chudleigh A, Hajarizadeh B, Martinello M, Adey S, Read P, Gilliver R, Applegate T, Treloar C, Maher L, Dore GJ (2019). Hepatitis C virus testing, liver disease assessment and direct-acting antiviral treatment uptake and outcomes in a service for people who are homeless in Sydney, Australia: The LiveRLife homelessness study. Journal of Viral Hepatitis.

[13] Bajis S, Grebely J, Hajarizadeh B, Applegate T, Marshall AD, Ellen Harrod M, Byrne J, Bath N, Read P, Edwards M, Gorton C, Hayllar J, Cock V, Peterson S, Thomson C, Weltman M, Jefferies M, Wood W, Haber P (2020). Hepatitis C virus testing, liver disease assessment and treatment uptake among people who inject drugs pre- and post-universal access to direct-acting antiviral treatment in Australia: The LiveRLife study. Journal of Viral Hepatitis.

[14] Castéra L, Vergniol J, Foucher J, Le Bail B, Chanteloup E, Haaser M, Darriet M, Couzigou P, De Lédinghen V (2005). Prospective comparison of transient elastography, Fibrotest, APRI, and liver biopsy for the assessment of fibrosis in chronic hepatitis C. Gastroenterology.

[15] Bush K, Kivlahan DR, McDonell MB, Fihn SD, Bradley KA (1998). The AUDIT alcohol consumption questions (AUDIT-C): an effective brief screening test for problem drinking. Ambulatory care quality improvement project (ACQUIP). Alcohol use disorders identification test. Archives of Internal Medicine.

[16] Bradley KA, DeBenedetti AF, Volk RJ, Williams EC, Frank D, Kivlahan DR (2007). AUDIT-C as a brief screen for alcohol misuse in primary care. Alcoholism, Clinical and Experimental Research.

[17] Bradley KA, Bush KR, Epler AJ, Dobie DJ, Davis TM, Sporleder JL, Maynard C, Burman ML, Kivlahan DR (2003). Two brief alcohol-screening tests from the alcohol use disorders identification test (AUDIT): Validation in a female veterans affairs patient population. Archives of Internal Medicine.

[18] Viney R, Norman R, King MT, Cronin P, Street DJ, Knox S, Ratcliffe J (2011). Time trade-off derived EQ-5D weights for Australia. Value Health.

[19] Belotti F, Deb P, Manning WG, Norton EC (2015). twopm: Two-part models. Stata Journal.

[20] Clemens S, Begum N, Harper C, Whitty JA, Scuffham PA (2014). A comparison of EQ-5D-3L population norms in Queensland, Australia, estimated using utility value sets from Australia, the UK and USA. Quality of Life Research.

[21] Fischer JA, Conrad S, Clavarino AM, Kemp R, Najman JM (2013). Quality of life of people who inject drugs: Characteristics and comparisons with other population samples. Quality of Life Research.

[22] Cossais S, Schwarzinger M, Pol S, Fontaine H, Larrey D, Pageaux G-P, Canva V, Mathurin P, Yazdanpanah Y, Deuffic-Burban S (2019). Quality of life in patients with chronic hepatitis C infection: Severe comorbidities and disease perception matter more than liver-disease stage. PLoS ONE.

[23] Han R, François C, Toumi M (2021). Systematic review of health state utility values used in European pharmacoeconomic evaluations for chronic hepatitis C: Impact on cost-effectiveness results. Applied Health Economics and Health Policy.

[24] Stone J, Artenie A, Hickman M, Martin NK, Degenhardt L, Fraser H, Vickerman P (2022). The contribution of unstable housing to HIV and hepatitis C virus transmission among people who inject drugs globally, regionally, and at country level: A modelling study. The Lancet Public Health.

[25] Cheng Q, Cunningham EB, Shih S, Amin J, Bruneau J, Artenie AA, Powis J, Litwin AH, Cooper C, Dalgard O, Hellard M, Bruggmann P, Marks P, Lacombe K, Stedman C, Read P, Hajarizadeh B, Dunlop AJ, Conway B (2023). Patient-reported outcomes during and after hepatitis c virus direct-acting antiviral treatment among people who inject drugs. Value Health.

[26] McDonald SA, Myring G, Palmateer NE, McAuley A, Beer L, Dillon JF, Hollingworth W, Gunson R, Hickman M, Hutchinson SJ (2023). Improved health-related quality of life after hepatitis C viraemic clearance among people who inject drugs may not be durable. Addiction.

[27] Aas CF, Vold JH, Skurtveit S, Lim AG, Ruths S, Islam K, Askildsen JE, Løberg E-M, Fadnes LT, Johansson KA, Aas CF, Buljovcic VB, Chalabianloo F, Daltveit JT, Alpers SE, Fadnes LT, Eriksen TF, Gundersen P, Hille V (2020). Health-related quality of life of long-term patients receiving opioid agonist therapy: A nested prospective cohort study in Norway. Substance Abuse Treatment, Prevention, and Policy.

[28] Feelemyer JP, Jarlais DCD, Arasteh K, Phillips BW, Hagan H (2014). Changes in quality of life (WHOQOL-BREF) and addiction severity index (ASI) among participants in opioid substitution treatment (OST) in low and middle income countries: An international systematic review. Drug and Alcohol Dependence.

[29] Nosyk B, Bray JW, Wittenberg E, Aden B, Eggman AA, Weiss RD, Potter J, Ang A, Hser YI, Ling W, Schackman BR (2015). Short term health-related quality of life improvement during opioid agonist treatment. Drug and Alcohol Dependence.

[30] Nosyk B, Guh DP, Sun H, Oviedo-Joekes E, Brissette S, Marsh DC, Schechter MT, Anis AH (2011). Health related quality of life trajectories of patients in opioid substitution treatment. Drug and Alcohol Dependence.

[31] Wang PW, Wu HC, Yen CN, Yeh YC, Chung KS, Chang HC, Yen CF (2012). Change in quality of life and its predictors in heroin users receiving methadone maintenance treatment in Taiwan: An 18-month follow-up study. American Journal of Drug and Alcohol Abuse.

[32] EuroQol--a new facility for the measurement of health-related quality of life*. Health Policy*, 1990. **16**(3): p. 199–208. 10.1016/0168-8510(90)90421-9 10109801

[33] Stark RG, Reitmeir P, Leidl R, König H-H (2010). Validity, reliability, and responsiveness of the EQ-5D in inflammatory bowel disease in Germany. Inflammatory Bowel Diseases.

[34] Short H, Al Sayah F, Ohinmaa A, Johnson JA (2021). The performance of the EQ-5D-3L in screening for anxiety and depressive symptoms in hospital and community settings. Health and Quality of Life Outcomes.

[35] Dyer MTD, Goldsmith KA, Sharples LS, Buxton MJ (2010). A review of health utilities using the EQ-5D in studies of cardiovascular disease. Health and Quality of Life Outcomes.

[36] Mafirakureva N, Dzingirai B, Postma MJ, van Hulst M, Khoza S (2016). Health-related quality of life in HIV/AIDS patients on antiretroviral therapy at a tertiary care facility in Zimbabwe. AIDS Care.

[37] Anderson RT, Baran RW, Dietz B, Kallwitz E, Erickson P, Revicki DA (2014). Development and initial psychometric evaluation of the hepatitis C virus-patient-reported outcomes (HCV-PRO) instrument. Quality of Life Research.

[38] Spaderna M, Kattakuzhy S, Kang SJ, George N, Bijole P, Ebah E, Eyasu R, Ogbumbadiugha O, Silk R, Gannon C, Davis A, Cover A, Gayle B, Narayanan S, Pao M, Kottilil S, Rosenthal E (2022). Hepatitis C cure and medications for opioid use disorder improve health-related quality of life in patients with opioid use disorder actively engaged in substance use. The International Journal on Drug Policy.

[39] Kåberg M, Larsson S, Bergström J, Hammarberg A (2023). Quality-adjusted life years among people who inject drugs in a needle syringe program in Sweden. Quality of Life Research.

[40] Stephenson J, Smith CM, Kearns B, Haywood A, Bissell P (2021). The association between obesity and quality of life: A retrospective analysis of a large-scale population-based cohort study. BMC Public Health.

