# Supplementary material for: Health-related quality of life among people who inject drugs in Australia
Source: Qual Life Res. 2023 Jun 23;32(11):3195–207. doi: 10.1007/s11136-023-03465-3 (PMC10522523; doi:10.1007/s11136-023-03465-3)
Supplement: Supplementary file 1 — Supplementary file1 (DOC 32 KB) [file 11136_2023_3465_MOESM1_ESM.docx]

**Appendix**

Table A1. Health-related quality of life by current treatment status among people who tested positive for HCV

|  | **n** | **Mean EQ-5D (SD)** | **Median EQ-5D (IQR)** | **p value*** | **Mean VAS (SD)** | **Median VAS (IQR)** | **p value*** |
| --- | --- | --- | --- | --- | --- | --- | --- |
| **HCV RNA positive** |  |  |  | 0.243 |  |  | 0.072 |
| No HCV treatment ever | 134 | 0.65 (0.24) | 0.69 (0.50 – 0.80) |  | 62 (25) | 68 (50 – 80) |  |
| Previous HCV treatment | 13 | 0.65 (0.28) | 0.73 (0.61 – 0.80) |  | 63 (26) | 75 (40 – 80) |  |
| Current HCV treatment | 26 | 0.72 (0.28) | 0.80 (0.62 – 1.00) |  | 73 (22) | 80 (65 – 90) |  |
| IQR, interquartile range  * Mann-Whitney U tests and Kruskal-Wallis tests were used to compare EQ-5D-3L and EQ-VAS scores between subgroups | | | | | | | |

Table A2. Health-related quality of life by current treatment status among people who self-reported current HCV infection

|  | **n** | **Mean EQ-5D (SD)** | **Median EQ-5D (IQR)** | **p value*** | **Mean VAS (SD)** | **Median VAS (IQR)** | **p value*** |
| --- | --- | --- | --- | --- | --- | --- | --- |
| **Self-report HCV infection** |  |  |  | 0.465 |  |  | 0.196 |
| No HCV treatment ever | 182 | 0.66 (0.26) | 0.73(0.50 – 0.80) |  | 60 (25) | 65 (50 – 80) |  |
| Previous HCV treatment | 37 | 0.64 (0.32) | 0.73 (0.50 – 0.80) |  | 64 (23) | 70 (50 – 80) |  |
| Current HCV treatment | 44 | 0.68 (0.31) | 0.80 (0.43 – 1.00) |  | 65 (29) | 73 (50 – 88) |  |
| IQR, interquartile range  * Mann-Whitney U tests and Kruskal-Wallis tests were used to compare EQ-5D-3L and EQ-VAS scores between subgroups | | | | | | | |

Table A3. Unadjusted analysis of factors associated with EQ-5D-3L scores among recent injectors (using two-part model)

|  | **Marginal effect** | **95% CI** | **p value*** |
| --- | --- | --- | --- |
| Age groups |  |  | 0.824 |
| 18-35 | - |  |  |
| 36-50 | -0.014 | -0.074, 0.046 | 0.644 |
| ≥ 51 | -0.040 | -0.111, 0.030 | 0.261 |
| Sex |  |  | 0.892 |
| Male | - |  |  |
| Female | -0.012 | -0.063, 0.040 | 0.660 |
| Aboriginal/Torres Strait Islander ethnicity |  |  | 0.699 |
| No | - |  |  |
| Yes | 0.001 | -0.055, 0.058 | 0.964 |
| Completed high school or higher education |  |  | 0.350 |
| No | - |  |  |
| Yes | 0.037 | -0.015, 0.088 | 0.161 |
| Main source of income |  |  | 0.006 |
| No income | - |  |  |
| Full-time/part-time/casual employment | 0.007 | -0.142, 0.157 | 0.922 |
| Government assistance | -0.131 | -0.257, -0.004 | 0.043 |
| Other | -0.190 | -0.357, -0.023 | 0.026 |
| Housing |  |  | 0.396 |
| Stable | - |  |  |
| Unstable | -0.034 | -0.084, 0.016 | 0.179 |
| Incarceration |  |  | 0.923 |
| Ever (not in past 12 months) | - |  |  |
| In past 12 months | -0.002 | -0.065, 0.061 | 0.951 |
| Never | 0.008 | -0.046, 0.062 | 0.767 |
| Hazardous alcohol consumption (AUDIT-C) |  |  | 0.320 |
| Never drinks | - |  |  |
| Low risk male/female | 0.025 | -0.034, 0.083 | 0.409 |
| High risk male/female | -0.030 | -0.086, 0.026 | 0.294 |
| Smoking stats |  |  | 0.361 |
| Never |  |  |  |
| Previous | -0.105 | -0.230, 0.019 | 0.097 |
| Current | -0.099 | -0.194, 0.003 | 0.043 |
| Current opioid agonist therapy |  |  | 0.386 |
| No | - |  |  |
| Yes | -0.009 | -0.059, 0.041 | 0.722 |
| HCV RNA test result |  |  | 0.867 |
| Negative | - |  |  |
| Positive | -0.005 | -0.055, 0.045 | 0.844 |
| Missing | 0.029 | -0.059, 0.117 | 0.519 |
| Self-reported HCV status |  |  | 0.527 |
| No | - |  |  |
| Yes | 0.023 | -0.034, 0.080 | 0.428 |
| Unknown | 0.034 | -0.036, 0.104 | 0.341 |
| FibroScan® liver disease staging |  |  | 0.345 |
| F0/F1 – No/mild fibrosis | - |  |  |
| F2/3 | -0.032 | -0.094, 0.029 | 0.305 |
| F4 – Cirrhosis | -0.007 | -0.107, 0.093 | 0.886 |
| Invalid score/missing | -0.095 | -0.231, 0.042 | 0.173 |
| *The overall p-values for variables with multiple categories were derived from Wald tests, using Stata function testparm. | | | |

Table A4. Adjusted multivariate analysis of factors associated with EQ-5D-3L scores among recent injectors (using two-part model)

|  | **Marginal effect** | **95% CI** | **p value*** |
| --- | --- | --- | --- |
| Main source of income |  |  | 0.005 |
| No income | - |  |  |
| Full-time/part-time/casual employment | 0.020 | -0.131, 0.171 | 0.797 |
| Government assistance | -0.121 | -0.250, 0.007 | 0.064 |
| Other | -0.188 | -0.358, -0.018 | 0.030 |
| Smoking stats |  |  | 0.231 |
| Never |  |  |  |
| Previous | -0.103 | -0.222, 0.017 | 0.092 |
| Current | -0.094 | -0.184, 0.003 | 0.043 |
| HCV RNA test result |  |  | 0.801 |
| Negative | - |  |  |
| Positive | -0.002 | -0.053, 0.048 | 0.925 |
| Missing | 0.025 | -0.063, 0.113 | 0.574 |
| FibroScan® liver disease staging |  |  | 0.232 |
| F0/F1 – No/mild fibrosis | - |  |  |
| F2/3 | -0.032 | -0.094, 0.029 | 0.082 |
| F4 – Cirrhosis | -0.014 | -0.122, 0.094 | 0.804 |
| Invalid score/missing | -0.077 | -0.209, 0.056 | 0.259 |
| *The overall p-values for variables with multiple categories were derived from Wald tests, using Stata function testparm. | | | |

Table A5. Unadjusted analysis of factors associated with EQ-5D-3L scores non-recent injectors (using two-part model)*

|  | **Marginal effect** | **95% CI** | **p value^** |
| --- | --- | --- | --- |
| Age groups |  |  | 0.608 |
| 18-35 | - |  |  |
| 36-50 | 0.015 | -0.071, 0.101 | 0.740 |
| ≥ 51 | 0.010 | -0.084, 0.103 | 0.840 |
| Sex |  |  | 0.703 |
| Male | - |  |  |
| Female | 0.009 | -0.058, 0.077 | 0.785 |
| Aboriginal/Torres Strait Islander ethnicity |  |  | 0.235 |
| No | - |  |  |
| Yes | -0.030 | -0.115, 0.055 | 0.493 |
| Completed high school or higher education |  |  | 0.226 |
| No | - |  |  |
| Yes | 0.025 | -0.042, 0.092 | 0.469 |
| Main source of income |  |  | 0.001 |
| No income | - |  |  |
| Full-time/part-time/casual employment | 0.137 | -0.025, 0.300 | 0.096 |
| Government assistance | -0.069 | -0.223, 0.084 | 0.374 |
| Other | -0.118 | -0.338, 0.102 | 0.294 |
| Housing |  |  | 0.598 |
| Stable | - |  |  |
| Unstable | -0.029 | -0.111, 0.052 | 0.482 |
| Incarceration |  |  | 0.720 |
| Ever (not in past 12 months) | - |  |  |
| In past 12 months | 0.032 | -0.062, 0.127 | 0.503 |
| Never | -0.001 | -0.073, 0.072 | 0.986 |
| Hazardous alcohol consumption (AUDIT-C) |  |  | 0.634 |
| Never drinks | - |  |  |
| Low risk male/female | 0.030 | -0.052, 0.112 | 0.469 |
| High risk male/female | 0.024 | -0.052, 0.100 | 0.534 |
| Smoking stats |  |  | 0.005 |
| Never |  |  |  |
| Previous | -0.020 | -0.172, 0.132 | 0.795 |
| Current | -0.078 | -0.193, 0.036 | 0.181 |
| Current opioid agonist therapy |  |  | 0.112 |
| No | - |  |  |
| Yes | -0.047 | -0.127, 0.034 | 0.253 |
| HCV RNA test result |  |  | 0.536 |
| Negative | - |  |  |
| Positive | 0.022 | -0.044, 0.089 | 0.509 |
| Missing | -0.044 | -0.161, 0.074 | 0.465 |
| Self-reported HCV status |  |  | 0.629 |
| No | - |  |  |
| Yes | -0.009 | -0.081, 0.064 | 0.817 |
| Unknown | -0.068 | -0.164, 0.028 | 0.167 |
| FibroScan® liver disease staging |  |  | 0.426 |
| F0/F1 – No/mild fibrosis | - |  |  |
| F2/3 | -0.077 | -0.166, 0.013 | 0.093 |
| F4 – Cirrhosis | -0.023 | -0.139, 0.094 | 0.703 |
| Invalid score/missing | -0.067 | -0.200, 0.067 | 0.328 |
| *Adjusted analysis was not conducted because there was no statistically significant factor in the unadjusted model  ^The overall p-values for variables with multiple categories were derived from Wald tests, using Stata function testparm. | | | |
